# Stage of estimated glomerular filtration rate (kidney function) is associated with greater prevalence and burden of aortic plaque: the Framingham heart study

**DOI:** 10.1186/1532-429X-17-S1-P417

**Published:** 2015-02-03

**Authors:** Michael L Chuang, Philimon N Gona, Noriko Oyama-Manabe, Daniel A Roseman, Gearóid M McMahon, Carol J Salton, Christopher J O'Donnell, Caroline S Fox, Warren J Manning

**Affiliations:** 1 NHLBI's Framingham Heart Study, Newton, MA, USA; 2 Beth Israel Deaconess Medical Center, Boston, MA, USA

## Background

Atherosclerotic aortic plaque (AAoP) is inversely associated with estimated glomerular filtration rate (eGFR), a commonly used continuous measure of kidney function. We sought to determine whether eGFR stage, categorical stratification by eGFR based on Kidney Disease: Improving Global Outcomes (KDIGO) criteria, is also associated with prevalence and burden of AAoP, as clinically patients are often characterized in terms of eGFR stage.

## Methods

We quantified prevalence and burden of AAoP in the abdominal aortas of 1690 adults (65±9y, 53% women) from the Framingham Offspring cohort who underwent cardiovascular magnetic resonance (CMR) and serum creatinine measured at the adjacent cycle exam. Abdominal imaging used a free-breathing, ECG-gated T2W axial black blood sequence with 5-mm slice thickness, 5-mm gap and 1.03x0.64 in-plane resolution. The eGFR was calculated from creatinine using Chronic Kidney Disease Epidemiology Collaboration (CKD-Epi) equations and study participants were stratified by eGFR stage: ≥90, 60-89, <60 ml/min/1.73m^2^. (As few participants had eGFR<60, we combined all of these participants into a single group, whereas KDIGO criteria further stratify patients.) We tested for trends (Cochrane-Armitage test) in prevalence and burden of AAoP by eGFR stage and used multivariable-adjusted logistic (plaque present or absent) and Tobit (plaque volume) regression models to assess association of AAoP with worsening eGFR stage.

## Results

Mean eGFR was 84±18 ml/min/1.73m^2^ in both sexes. Table 1 shows prevalence and burden of AAoP by sex and eGFR stage. On MV-adjusted logistic regression greater age (odds ratio, OR=1.32/10 years, p<0.0001), fasting glucose (OR=1.10/10mg/dL, p=0.006), pack-years smoked and current smoking (OR=1.66, p=0.007), cholesterol treatment (OR=1.40, p=0.017) and eGFR stage (OR=1.37 per stage, p=0.001) were associated with prevalent AAoP. With Tobit modeling only greater age (hazard ratio, HR=1.23/10 years, p=0.0002, log pack-years smoked (HR=1.06, p<0.0001) and eGFR stage (HR=1.24, p=0.005) were associated with greater amount of AAoP.

**Table 1:** Prevalence and burden of aortic plaque by eGFR stage.

eGFR	N (%)	Prevalent AAoP	AAoP Volume, cm3	N (%)	Prevalent AAoP	AAoP Volume, cm3
	Men			Women		

≥90	309 (39.1%)	115 (37.2%)	0.27±0.85	358 (39.8%)	152 (42.5%)	0.36±0.87

60-89	411 (52.0%)	207 (50.4%)	0.60±1.55	455 (50.6%)	237 (52.1%)	0.55±1.20

<60	70 (8.9%)	49 (70.0%)	1.88±2.91	87 (9.7%)	54 (62.1%)	1.16±2.98

P for trend		<0.0001	<0.0001		0.0002	<0.0001

## Conclusions

In a community-dwelling cohort of middle aged and older aduts, reduced kidney function, as assessed by eGFR and KDIGO stage is associated with greater prevalence of and volume of AAoP, even after adjustment for other cardiovascular disease risk factors.

## Funding

Supported by the National Heart, Lung and Blood Institute's Framingham Heart Study (N01-HC-25195), and by RO1 HL70279.

